# Algorithm of traumatic brain injury management at Indonesia in the COVID 19 pandemic ERA. Retrospective cohort study

**DOI:** 10.1016/j.amsu.2021.01.008

**Published:** 2021-01-16

**Authors:** Rohadi Muhammad Rosyidi, Dewa Putu Wisnu Wardhana, Tedy Apriawan, Asra Al Fauzi, Bambang Priyanto, Kevin Gunawan, Setyo Widi Nugroho, Krisna Tsaniadi Prihastomo, Muhammad Deni Nasution, Andi Ihwan

**Affiliations:** a Department of Neurosurgery, Medical Faculty of Mataram University, West Nusa Tenggara General Hospital, Mataram, Indonesia; b Department of Neurosurgery,Udayana University Hospital, Medical Faculty of Udayana University, Bali, Indonesia; c Department of Neurosurgery, Medical Faculty of Airlangga University, Dr. Soetomo General Hospital Medical Center, Surabaya, East Java, Indonesia; d Department of Neurosurgery, Medical Faculty of Indonesia University, Cipto Mangunkusumo National General Hospital, Jakarta, Indonesia; e Department of Neurosurgery, Dr. Kariadi General Hospital Medical Center, Semarang, Center Java, Indonesia; f Department of Neurosurgery, Medical Faculty of Sumatera Utara University, Medan, North Sumatera, Indonesia; g Department of Neurosurgery, Medical Faculty of Hasanuddin University, Makassar, South Sulawesi, Indonesia

**Keywords:** Covid 19, Traumatic brain injury, Management of neurotrauma

## Abstract

**Objectives:**

COVID-19, a global pandemic, affects neurosurgical care in Indonesia. This study has objective to propose guideline and algorithm recommendation for the management of TBI patients during this pandemic, which can be used flexibly at neurosurgery centers, both in Indonesia and throughout the world.

**Methods:**

We performed retrospective Cohort analysis from TBI database at tertiary public general hospitals. All neurotrauma cases from mid-February until mid-August 2020 was included in this study. The chronology of COVID-19 pandemics impact in Indonesia was defined by early period from mid-February until end of May 2020, and late period are latter. All subjects undergone the screening and perioperative measures that based on our proposes scoring system and algorithm as follows.

**Results:**

There are many guidelines that explain screening methods in neurosurgery patients in general, as well as neurotrauma in particular. But here, we proposed our own scoring and screening algorithm that has been developed based on conditions in Indonesia. In total of 757 neurotrauma cases data were collected from the pandemic starts in Indonesia.

**Discussion:**

Screening is a crucial initial step in this pandemic period, not only for COVID patients, but also all patients who enter the emergency room. The use of PPE is a necessity in several neurosurgery centers, especially with high COVID-19 case rates.

**Conclusion:**

The management of neurotrauma patients with suspected and confirmed COVID-19 requires special attention, starting from admission of the patient in ER. Rapid scoring and screening are important and the highest level of PPE is mandatory during patient care.

## Highlights

• Retrospective analysis from TBI database at tertiary public general hospitals in Indonesia
• This study has objective to propose guideline and algorithm recommendation for the management of TBI patients during this pandemic
• Total of 757 neurotrauma cases data were collected from the pandemic starts in Indonesia
• Screening is a crucial initial step in this pandemic period, not only for COVID patients, but also all patients who enter the emergency room
• The management of neurotrauma patients with suspected and confirmed COVID-19 requires special attention, starting from admission of the patient in ER

## 1 Introduction

In December 2019, a new type of corona virus was discovered as a cause of pneumonia, which became known as SARS-CoV 2. The disease became known as COVID-19 [1]. Finally, on March 11, 2020 WHO declared COVID-19 as a global pandemic [2]. As of July 22nd, 2020, a total of 14 765 256 people had been reported confirmed for coronavirus disease (COVID-19) globally. Among these, there have been 612 054 deaths reported related to COVID-19. As of July 22nd, 2020, the Government of the Republic of Indonesia has reported 91751 confirmed cases and 4459 deaths related to COVID-19, while 50255 patients have recovered from the disease. Cases in Indonesia are mainly centered on big cities (municipalities and provincial capitals) in Indonesia, which are also the place for neurosurgical referral centers for their respective regions [3].

Traumatic brain injury (TBI) is one of the main causes of death, not only in the lower- and middle-income countries, but also in high-income countries. TBI is also associated with high morbidity and mortality rates and high treatment costs. Therefore, management of TBI cases, especially in emergency rooms, is very important and crucial to determine patient outcomes [4,5].

In this pandemic condition, the number of TBI cases has indeed decreased significantly [6]. But after the start of new normal in Indonesia, traffic flow began to return to normal and increase the number of TBI cases in the neurosurgery centers. Unfortunately, this was not followed by a decrease in the number of COVID-19 cases in Indonesia which continued to increase significantly, thereby increasing the possibility of neurosurgeons, both residents and senior neurosurgeons, being exposed to COVID-19 disease. Therefore, a guideline and algorithm recommendation for the management of TBI patients during this pandemic are needed, which can be used flexibly at neurosurgery centers, both in Indonesia and throughout the world.

## 2 Methods

We performed retrospective cohort analysis from TBI database at tertiary public general hospitals at Jakarta, Medan, Surabaya, Semarang, Denpasar and Mataram City, according to The STROCSS 2019 Guideline [7]. All neurotrauma cases from mid-February until mid-August 2020 was included in this study. Pediatric age TBI group was defined under the age of 18. The chronology of COVID-19 pandemics impact in Indonesia was defined by early period from mid-February until end of May 2020, and late period are latter. All subjects undergone the screening and perioperative measures that based on our proposes scoring system and algorithm as follows. The statistical analysis was performed using Statistical Package for the Social Sciences (SPSS) version 25 for Macintosh. Descriptive analysis, independent sample T-test and correlation test was performed on the dataset. Final at was summarized in tables and graph.

## 3 Preoperative preparation

### 3.1 Screening

Screening is a crucial initial step in this pandemic period, not only for COVID patients, but also all patients who enter the emergency room. During a pandemic, all patients must undergo COVID-19 screening and be treated as a suspected COVID-19 until proven different [4,8].

The first thing to note is the security of the medical staff who will handle patients in the emergency room. The use of PPE level III is a necessity in several neurosurgery centers, especially with high COVID-19 case rates [6,8]. However, some centers and guidelines allow the use of PPE level II [6].

**Fig. 1:**
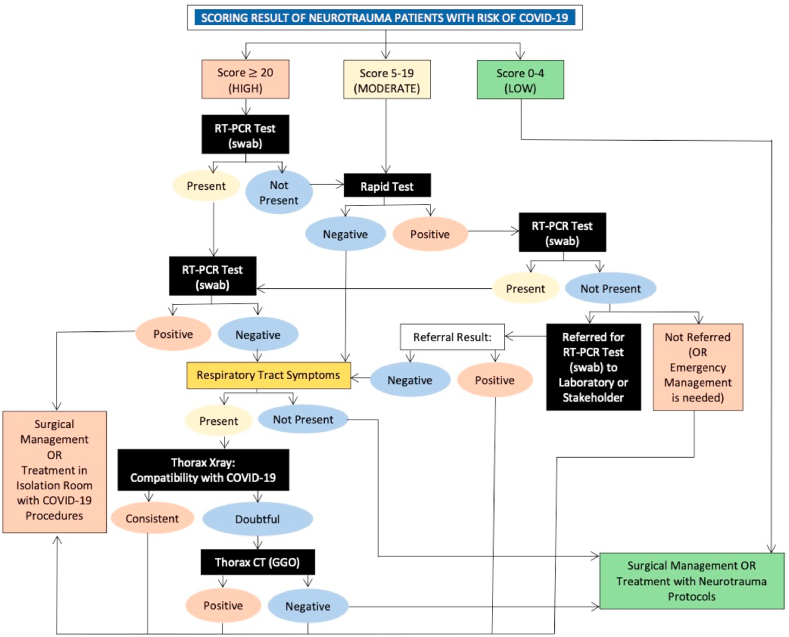
Proposed screening algorithm for neurotrauma patients with risk of COVID-19.

Next up is screening for COVID-19 patients who enter the emergency room. There are many guidelines that explain screening methods in neurosurgery patients in general, as well as neurotrauma in particular [6,9]. But here, we proposed our own scoring and screening algorithm that has been developed based on conditions in Indonesia (Table 1 and Fig. 1).

**Table 1:** Proposed COVID-19 scoring for neurotrauma patients in emergency room.

No	Subjective/objective Findings	Scoring Criteria	Score
MAJOR			
1	History of contact with Confirmed COVID-19 patients without standardized PPE + ≥ 1 minor objective findings	If Major Criteria 1–3 are met 1 or more, score = 20	-
2	CXR: Bilateral Basal Consolidation		
3	Thorax CT-Scan Findings: GGO Bilateral		
MINOR (Subjective)			
1	Work/attend mass gatherings/places of worship/social gathering/parties/markets or service places (airports, banks, etc.)	If minor criteria number 1–4 are met 1 or more, score = 4	-
2	Live or travel in an infected area/community (domestic and foreign)		
3	Family (1 house) works or travels to a place with positive/risky cases		
4	The surrounding environment obtained a confirmation case		
MINOR (Objective)			
5	Fever/history of fever in the last 14 days (≥37,5)	If minor criteria number 5–8 are met 1 or more, score = 4	-
6	Anosmia		
7	Gastrointestinal symptoms (diarrhea, nausea, vomiting, abdominal pain)		
8	Respiratory symptoms (cough, flu, dyspneu)		
9	Comorbidity factors (DM/HT/CKD/Malignancy/autoimmune/cardiac problem/obesity/pregnancy)	1	-
10	Leukopenia (<5000)	1	-
11	NLR >3,5	1	-
12	ALC <1100	1	-
13	Trombositopenia (<180.000)	1	-
14	Increased CRP (>5x normal)	1	-
15	CXR: bilateral consolidation (basal 1 - peripheral)	1	-
16	CXR: bilateral diffuse consolidation	1	-
17	CXR: unilateral consolidation	1	-
18	CXR: central bilateral consolidation	1	-
19	A history of contact with COVID-19 patients was confirmed (without standard PPE) without other findings	10	-
**TOTAL**			-
	Score 1–4: Low risk	Low/Moderate/high risk	
	Score 5–19: Moderate risk		
	Score ≥ 20: High risk

Scoring is done by doing history taking, physical examination and basic laboratory studies, such as complete blood test and CRP. A basic radiological examination of the chest x-ray was also performed to add an assessment of the rapid scoring of COVID-19 in patients with emergency conditions. This scoring makes it easier for examiners to objectively assess the possibility of COVID-19 in patients while carrying out investigations that are also essentially needed in an emergency room [10].

After scoring, screening of the patient the proceed with the algorithm below. The algorithm has been adjusted to the real clinical conditions in Indonesia, bearing in mind that not all hospitals have facilities to perform advanced diagnostic tests or RT-PCR tests for COVID-19 patients.

Examination of the algorithm above is done in stages, starting with checks with low sensitivity and specificity to the high. Rapid test examination is still being done, although from WHO and most studies show that the use of rapid tests cannot be used as a basis for determining clinical action (WHO). Even so studies show that rapid tests can still be used as a screening tool and rapid triage for individuals suspected of COVID-19 in communities that may be infected [11].

Chest X-ray examination is also still included as one of the COVID-19 assessment criteria for scoring and screening. This examination, in addition to being easy, inexpensive and fast, is also still reliable enough to be one of the COVID-19 detection devices with a sensitivity ranging from 68%. It should be noted that chest X-ray images in COVID-19 patients can vary, but the majority of patients show basal, peripheral and bilateral predominance [12].

Thorax CT Scan examination is the most specific and sensitive examination to assess COVID-19 status in patients. The specificity and sensitivity of CT scans for diagnosing COVID-19 ranges above 90% in the average study [13,14]. Nevertheless, Thorax's CT Scan is still limited for identifying specific viruses and distinguishing between viruses [14,15]. In addition, because the Thorax CT Scan is relatively expensive and has not been distributed in all hospitals in Indonesia, so this examination does not become our routine examination on the algorithm above. Although at all neurosurgery centers in Indonesia, CT scans are available making it possible to do outside of financing problems.

RT-PCR examination is a routine examination conducted at COVID-19 referral hospitals because it is sufficiently specific and sensitive to assess the status of COVID-19 [16]. Unfortunately, in Indonesia, most hospitals have not been able to carry out this examination so that the algorithm above RT-PCR can be done by referring to other hospitals.

### 3.2 Patient transfer

Transfer of patients from the emergency room to the ward or operating room still requires special protection, especially for suspected or confirmed cases. Special transfer paths should be applied for patients with suspected or confirmed cases to avoid contamination. A minimum PPE level 2 is recommended in this condition [6,8].

The use of consumables and special transportation also needs to be implemented. Sterilization with disinfectant liquid for tools that are used repeatedly such as beds, oxygen cylinders, etc. should not be forgotten and regulated in the policies of each hospital [6].

### 3.3 Operating room preparation

For COVID-19 confirmed cases, a negative-pressure operating room must be used. The operating room must be disinfected before and after use with disinfectant solutions. Disposable surgery material is used for patients who are confirmed to be COVID-19 [4,6,8].

## 4 Operative considerations

The operation is carried out with careful preparation and with the shortest possible duration of time with a minimum possible number of personnel. Therefore surgeons, anesthesiologists and experienced operating room staff are needed to shorten the duration of the operation and minimize the personnel used. This is done to minimize the risk of exposure. The strict use of level III PPE must be used for surgery of confirmed or suspected COVID-19 patients [4,17].

## 5 Post-operative management

### 5.1 Operating rooms and operating room personnel

The operating room must be thoroughly disinfected with disinfectant fluid for at least 30 min. Anesthesia instruments, in addition to spray disinfection, are also wiped with a special cloth to sterilize them. Conditions of special operating room air pressure COVID-19, ventilation and air conditioning also need to be considered [6,8].

Disinfection of operating room personnel is also important. It is important to remove APD level II in a special room and dispose of it in a special bin. Handling of medical waste that is used when in contact with a suspect or confirmed patient COVID-19 also needs to be considered [6,8,18].

### 5.2 Transfer and care of patients in the room

Transfers of patients with suspected COVID-19 from the operating room to the treatment room must use level III PPE [6]. The use of special transfer equipment for patients suspected of COVID-19 as in the preoperative transfer was also carried out, as well as the disinfection of the transportation equipment [6,8].

Treatment of suspected and confirmed patients with COVID-19 is carried out in a special room with negative pressure, especially for patients who are confirmed. The use of PPE level III is again emphasized to always be used in any condition when dealing with COVID-19 patients. A minimum PPE level I can be used in a non-COVID patient room [6].

The use of disposable medical tools and products is still applied for suspected patients and COVID-19 confirmed even in the treatment room. Patients with COVID-19 need to be confirmed to be negative, with at least 2x negative swabs when they are discharged, and also isolated 14 days postoperatively in the special ward [6,8]. The protocol for handling COVID-19 in each hospital needs to be evaluated in accordance with the conditions and availability of facilities and infrastructure, but must not ignore the recommendations of the existing guidelines [4,[19], [20], [21]].

## 6 Results

In total of 757 neurotrauma cases data were collected from the pandemic starts in Indonesia from mid-February until mid-August 2020. Most of them are from Denpasar city; followed by Jakarta city, Medan city Semarang city, Mataram city, and Surabaya city. The TBI cases was predominantly male, and it is consistent in all cities. In this study, the age distribution of subjects are from 1 month to 94 years old, mean age was 35.15 years old and median age was 32 years old. From the early period of COVID-19 pandemic in Indonesia, the total number of TBI case was 468 cases (61.8%) and become 289 cases (38.2%). The characteristic of the subjects are summarized in Table 2.

**Table 2:** Characteristic of traumatic brain injury in Indonesia.

Characteristic	Number of Patients (%)
Total patients (N)	757
Males	570 (75.3%)
Females	187 (24.7%)
Age group	
Pediatric	160 (21.1%)
Other	597 (78.9%)
Month	
Mid-February	83 (11.0%)
March	135 (17.8%)
April	117 (15.5%)
June	133 (17.6%)
July	121 (16.0%)
Mid-August	48 (6.3%)
City	
Denpasar	273 (36.1%)
Jakarta	178 (23.5%)
Medan	89 (11.8%)
Semarang	85 (11.2%)
Mataram	79 (10.4%)
Surabaya	53 (7.0%)
Therapy	
Operation	273 (36.1%)
Conservative	484 (63.9%)
COVID-19 TBI score	
Low risk	565 (74.6%)
Moderate risk	189 (25.0%)
High risk	3 (0.4%)
Final COVID-19 status^a^	
Confirmed	3 (0.4%)
Negative	754 (99.6%)

a Final status of COVID-19 infection was obtained from RT-PCR examination.

In this study base on Table 3 and Table 4, we revealed small number of patients (0.4%) that considered as high risk being infected by COVID-19 virus. Therefore, in order to measure the significance of the scoring system and to prevent bias, the last two risk categories on scoring interpretation was merged. There are three confirmed TBI cases with associated COVID-19 infection (0.4%). All of those cases are adults, already present with moderate-severe risk and come at latter period (Table 4).There was significant (P 0.003) difference between the groups (low, moderate-high risk) of scoring system with the final COVID-19 status in our subjects with the correlation coefficient 0.108.

**Table 3:** Mean COVID-19 TBI score on early and late period of pandemics based on Cities grup, Age Grup, and Therapy.

	Chronology	
	Early period	Late period
	Mean COVID-19 TBI Score ±SD	Mean COVID-19 TBI Score ±SD
City		
Denpasar	1.60 ± 1.95	1.51 ± 1.86
Jakarta	5.48 ± 2.49	3.15 ± 2.07
Medan	6.32 ± 3.47	5.85 ± 2.37
Semarang	0 ± 0	1.00 ± 2.45
Mataram	1.30 ± 1.73	1.23 ± 1.96
Surabaya	2.85 ± 3.18	6.74 ± 4.61
Age group		
Pediatric	2.66 ± 2.55	2.21 ± 2.30
Other	2.76 ± 3.20	3.18 ± 3.26
Therapy		
Operation	2.06 ± 2.58	2.67 ± 2.97
Conservative	3.15 ± 3.26	3.13 ± 3.17

**Table 4:** The final COVID-19 status of the TBI patients based on Chronology, Age Groups, and Risks.

	Final COVID-19 status				
	Confirmed		Negative		P value^∗∗^
	Number of patients (%)	Mean ± SD	Number of patients (%)	Mean ± SD	
Chronology					
Early period	0 (0%)	-	468 (62.1%)	2.74 ± 3.07	0.000
Late period	3 (100%)	14.00 ± 12.17	286 (37.9%)	2.86 ± 2.72	
Age group					0.014
Pediatric	0 (0%)	-	160 (21.2%)	2.49 ± 2.46	
Other	3 (100%)	14.00 ± 12.17	594 (78.8%)	2.87 ± 3.05	
COVID-19 TBI score					0.003
Low risk	0 (0%)	-	565 (74.9%)	1.56 ± 1.84	
Moderate-high risk	3 (100%)	14.00 ± 12.17	189 (25.1%)	6.44 ± 2.56	

	Final COVID-19 status^∗∗∗^	
	P value	Correlation coefficient
Chronology		−0.080
Early period	0.027*	
Late period		
Age group	0.370	−0.033
Pediatric		
Other		
COVID-19 TBI score	0.003*	−0.108
Low risk		
Moderate-high risk		

*significant with p < 0.05.

**Independent sample T test

***2 tailed correlation test.

## 7 Discussion

Rapid scoring and screening are important for assessing the risk status of COVID-19 patients, starting from admission of the patient in ER. Because the patient's COVID-19 status often cannot be ruled until the patient needs surgery or treatment, the use of level III APD, routine disinfection and the use of disposable medical devices and materials is important to be applied when managing patients with suspected or confirmed COVID-19. The COVID-19 protocol in every hospital can be different, but safety standards for medical staff and other patients should not be lowered under any circumstances.

Our scoring system result is related with the end of patient's COVID-19 status (p. 0.003), however the correlation is still weak. That may because our database number is relatively less, or that results from the strict new normal concept in societies where the people adapt to infection prevention habit.

This study strength: we only include TBI cases from public general hospitals where the protocols and best standard of care are well maintained. However this study also has weakness that the number of TBI cases with positive COVID-19 confirmed case are low. Therefore, prospective study with larger number of subjects need to be conducted to measure the accuracy for the scoring screening instrument.

The limitation of this study is that this algorithm is used in areas with limited facilities and in conditions of limited PCR and rapid test facilities. not all regions in Indonesia with ideal facilities, in contrast to developed countries which have complete facilities and covid test kits that are complete and free by the government.

Compared to other published algorithms, generally they are from countries with complete Covid 19 PCR therapy and diagnostic facilities. In the algorithm that we present for modification in areas with limited facilities, it is necessary to adjust to the available resources. We have made this manuscript by adjusting our conditions in regions in Indonesia, adjusting also to the limited conditions of therapeutic and diagnostic facilities in Indonesia. In this manuscript we also report the number of brain injury patients treated in a multicentre in a number of cities in Indonesia.

## 8 Conclusion

The management of neurotrauma patients with suspected and confirmed COVID-19 requires special attention. Our proposed algorithm of traumatic brain injury management in Indonesia are considered to be useful and applicable in order to stratified the risk of COVID-19 infection especially in countries with limited facilities.

## Disclosures

The authors report no conflict of interest.

## Provenance and peer review

Not commissioned, externally peer-reviewed.

## Funding

No funding or sponsorship.

## Ethical approval

All procedure for research has been approved by Ethics Commission Faculty of Medicine, Mataram University.

## Consent

This manuscript data from medical record patient with traumatic brain injury during pandemic in multi center hospital in indonesia.

## Author contribution

RHA, NUW, TDY, UZI, BAM, KVG, SWN, NAA, MDN, and AIH wrote the manuscript and participated in the study design. RHA, NUW, TDY, UZI, BAM, KVG, SWN, NAA, MDN, and AIH drafted and revised the manuscript. RHA, NUW, TDY, UZI, BAM, KVG, SWN, NAA, MDN, and AIH performed head trauma treatment and surgery. RHA, NUW, TDY, UZI, BAM, KVG, SWN, NAA, MDN, and AIH performed bioinformatics analyses and revised the manuscript. All authors read and approved the final manuscript.

## Registration of research studies

1. Name of the registry: http://www.researchregistry.com. Registration Date: January 05, 2021 05:30
2. Unique Identifying number or registration ID: researchregistry6419
3. Hyperlink to your specific registration (must be publicly accessible and will be checked): https://www.researchregistry.com/register-now#user-researchregistry/registerresearchdetails/5ff3f967d01bdc001bd9e8bc/

## Guarantor

Rohadi Muhammad Rosyidi.

## Declaration of competing interest

The authors declare that they have no conflict of interests.
